# OCT Imaging in Murine Models of Alzheimer’s Disease in a Systematic Review: Findings, Methodology and Future Perspectives

**DOI:** 10.3390/biomedicines12030528

**Published:** 2024-02-27

**Authors:** Lidia Sánchez-Puebla, Inés López-Cuenca, Elena Salobrar-García, Ana I. Ramírez, José A. Fernández-Albarral, José A. Matamoros, Lorena Elvira-Hurtado, Juan J. Salazar, José M. Ramírez, Rosa de Hoz

**Affiliations:** 1 Ramon Castroviejo Institute for Ophthalmic Research, Complutense University of Madrid, 28040 Madrid, Spain; lidsan02@ucm.es (L.S.-P.); inelopez@ucm.es (I.L.-C.); elenasalobrar@med.ucm.es (E.S.-G.); airamirez@med.ucm.es (A.I.R.); joseaf08@ucm.es (J.A.F.-A.); jomatamo@ucm.es (J.A.M.); marelvir@ucm.es (L.E.-H.); jjsalazar@med.ucm.es (J.J.S.); 2 Health Research Institute of the Hospital Clínico San Carlos (IdISSC), 28040 Madrid, Spain; 3 Department of Immunology, Ophthalmology and ENT, Faculty of Optics and Optometry, Complutense University of Madrid, 28040 Madrid, Spain; 4 Department of Immunology, Ophthalmology and ENT, School of Medicine, Complutense University of Madrid, 28040 Madrid, Spain

**Keywords:** Alzheimer’s disease, AD mouse model, optical coherence tomography, retina

## Abstract

The murine models of Alzheimer’s disease (AD) have advanced our understanding of the pathophysiology. In vivo studies of the retina using optical coherence tomography (OCT) have complemented histological methods; however, the lack of standardisation in OCT methodologies for murine models of AD has led to significant variations in the results of different studies. A literature search in PubMed and Scopus has been performed to review the different methods used in these models using OCT and to analyse the methodological characteristics of each study. In addition, some recommendations are offered to overcome the challenges of using OCT in murine models. The results reveal a lack of consensus on OCT device use, retinal area analysed, segmentation techniques, and analysis software. Although some studies use the same OCT device, variations in other parameters make the direct comparison of results difficult. Standardisation of retinal analysis criteria in murine models of AD using OCT is crucial to ensure consistent and comparable results. This implies the application of uniform measurement and segmentation protocols. Despite the absence of standardisation, OCT has proven valuable in advancing our understanding of the pathophysiology of AD.

## 1. Introduction

Alzheimer’s disease (AD) is considered the leading cause of dementia (up to 70% of all cases) and it is believed that up to 152 million people will be affected by 2050 [1]. The pathophysiology of the disease is characterised by abnormal processing and the clearance of two proteins, amyloid-beta (Aβ) and phosphorylated tau (p-Tau), which are believed to lead to a neurotoxic inflammatory process, neuronal dysfunction, and ultimately neurodegeneration [2,3,4]. The disease begins up to 20 years before cognitive signs appear and this period is critical in the search for early diagnosis and future treatment [5].

Despite the extensive knowledge we already have of the pathology, there are still many histopathological aspects that cannot be studied in patients. This problem, added to the establishment of preclinical stages of the disease [6] makes the use of murine models necessary and very useful [7]. Due to this need for study, murine models of the disease have evolved into humanised models, expressing characteristics similar to those found in patients with the disease in its different stages. These models help to better understand the evolution of the disease [8].

**Figure 1:**
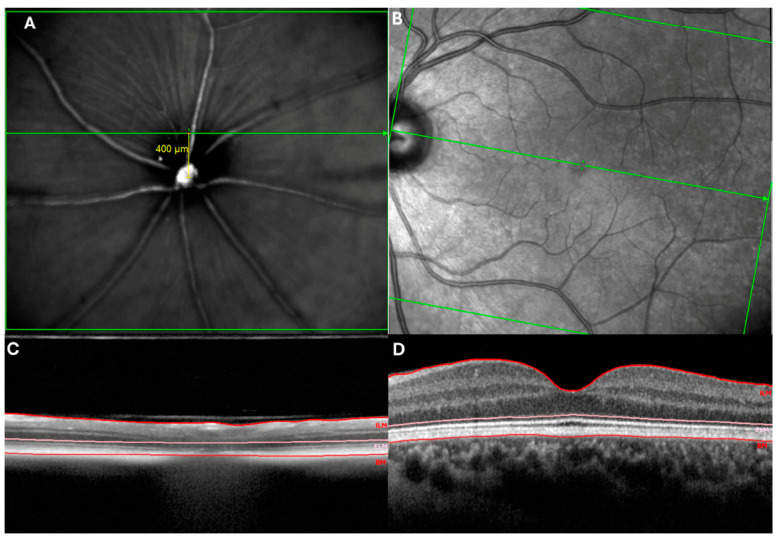
Analysis of mouse and human retina by optical coherence tomography (OCT). (**A**) HRA fundus image of mouse retina. The yellow line marks the retinal area with the highest concentration of photoreceptors, located dorsally 400 μm from the centre of the optic nerve being located dorsally 400 μm from the centre of the optic nerve (**B**) HRA fundus image of human retina. (**C**) Cross-sectional OCT scans showing the inner and outer layers in mouse retina. (**D**) Cross-sectional OCT scans showing the inner and outer layers in human retina.

Unlike humans, mouse retinas do not have a distinct macular area [9] and the optic disc is located in the geometric centre of the retina. The retinal zone corresponding to the macular area has been considered a region that has a higher concentration of photoreceptors, being located dorsally 400 μm from the centre of the optic nerve head (10° and 20° of eccentricity). Moreover, this location coincides with the optic axis of the mouse eye [10]. Regarding photoreceptors, in humans the maximum density is found in the macular area giving us a high spatial resolution. Interestingly, the values reported by Carter-Dawson and LaVail [11] and Jeon et al. [12] on the mouse retina show that the central retina of these rodents has a higher concentration of photoreceptors compared to those in cats or in humans. Therefore, despite differences in anatomy, the mouse retina has a high similarity to the human retina [9] (Figure 1). In addition, the retina of murine models of AD shows characteristics similar to those found in the AD human brain, e.g., an accumulation of Aβ oligomers, increased p-Tau, and neurodegeneration [13].

In patients with AD, structural changes in the thickness of different retinal layers in the macular area and in the thickness of the retinal nerve fibre layer (RNFL) in the peripapillary area have also been reported [14,15,16,17]. For this reason, retinal analysis by optical coherence tomography (OCT) has been proposed as a good biomarker for early detection and monitoring of AD-related changes in the central nervous system [18,19]. However, the findings found do not appear homogeneous, and in many cases contradict each other [20,21]. This is because confounding factors such as age and different disease stages cannot be easily eliminated from the quantitative retinal measurement, limiting the reliable interpretation of changes associated with AD [22]. The examination protocols differ greatly depending on the commercial OCT devices and are not comparable due to the resolution, the segmentation of the retina, and the sectors analysed [23]. In the preclinical stages of the disease, where subjects show positive disease biomarkers and normal cognition, functional and structural ophthalmological changes are already found [24]. Therefore, oculomics opens the opportunity to monitor and diagnose, before clinical manifestations appear, both systemic diseases (such as diabetes, arterial hypertension, and sarcopenia) and neurodegenerative diseases (AD, Parkinson’s disease, or multiple sclerosis), thus improving quality of life of patients [25,26].

To better understand the pathogenesis of AD in the retina, transgenic murine animal models have been used to analyse the deposits of Aβ plaques and neurofibrillary tangles or the neurodegeneration that occur in the retina [27]. Histological evaluation has contributed to understanding the mechanisms and pathophysiology of the disease [28], but with the advance of technology, OCT has become one of the techniques currently used because it allows in vivo analysis of the retina and thus enables the analysis of the model at different time points, allowing longitudinal studies to control the disease progression and follow-up with the secondary therapeutic effects [29]. In addition, the latest generation OCT offers advantages such as a high image resolution comparable to histological images offered by high-end microscopes [30] and the possibility of obtaining volumetric data of structural changes, providing better supplementary information than the visualisation the images obtained by microscopy [31]. There are few OCTs adapted to the eye of murine models, and in many cases, devices manufactured or modified by the researchers themselves are used [32]. As with clinical OCT, data collection protocols are not homogeneous and there is no consensus as to which areas are the most important for this analysis.

Therefore, the aim of the present work is to review the different OCT analysis methods that have been carried out in murine models of AD and to analyse what type of OCT has been used, which retinal sectors have been analysed, as well as the retinal layer segmentation and which software has been used for that. In addition, we provide a series of recommendations to overcome the challenges encountered in the use of OCT imaging of murine models of AD.

## 2. Materials and Methods

### 2.1. Search Strategy

We performed the literature search up to April 2023 using “MESH” terms in PubMed and Scopus. This review was performed in accordance with the PRISMA 2020 Statement guidelines, and it was registered in Insplay (INPLASY202410073). The terms used were: “Alzheimer’s mouse model”, “Optical Coherence Tomography”, “retina” as well as their combinations.

**Figure 2:**
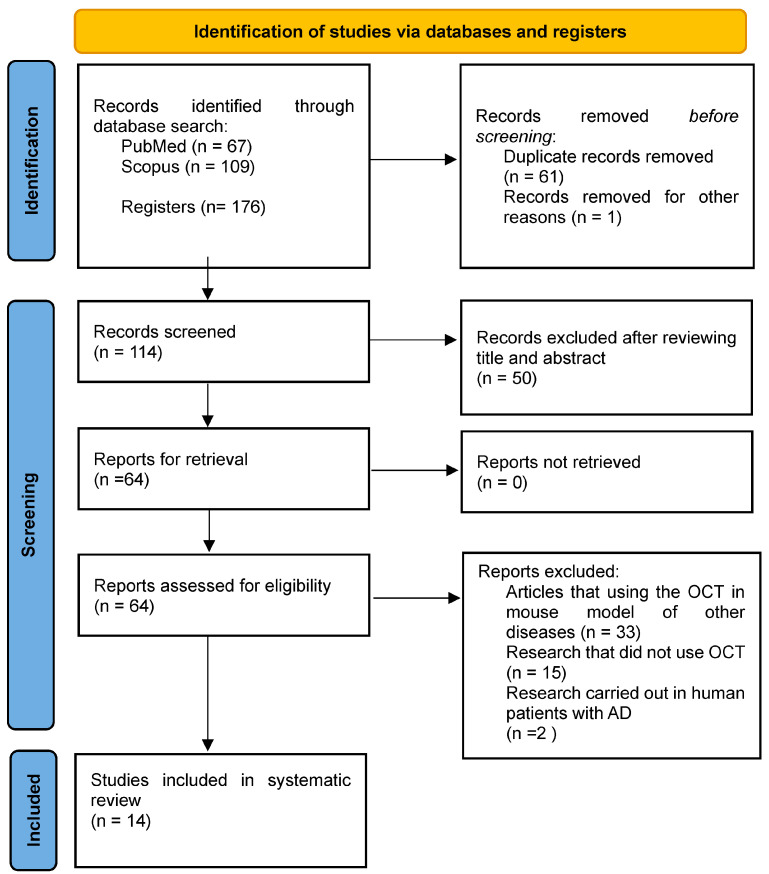
Flowchart study selection process according to the PRISMA statement.

The search resulted in indexed articles (67 and 109 references from PubMed and Scopus, respectively). After removing duplicates (n = 62), a total of 114 articles were analysed. After screening of titles and abstracts, 50 studies were excluded and only 64 full-text studies were retrieved and assessed for final eligibility. In addition, 50 articles were excluded for the following reasons: articles using OCT in mouse models of other diseases (n = 33); papers not using OCT (n = 15); research in human AD patients (n = 2). Finally, 14 studies were included in the systematic review (Figure 2).

### 2.2. Inclusion and Exclusion Criteria

We filtered the articles by author criteria; the terms had to be in the title, in the abstract, or in the article and they should be written in English or Spanish. These papers should clearly explain the OCT analysis techniques that were carried out on an animal model of AD. In addition, we considered articles that would help us better understand the anatomy of the mouse retina, as well as the use of murine retinas in disease research.

Inclusion criteria: we considered articles according to the following criteria: (i) articles about the anatomy of mouse retina; (ii) research that performed OCT in AD mouse models; (iii) research that performed OCT in combination with other technologies.

Exclusion criteria: we excluded the studies with the following characteristics: (i) articles that used the OCT in mouse models for other diseases (retinal degenerations, genetic diseases, or systemic diseases affecting the retina); (ii) research that did not use OCT; (iii) research carried out in human patients with AD.

## 3. Results

### 3.1. Mouse Model Analysed

Several studies have investigated the retina of murine models of AD using OCT in the past decade. The most analysed models include *TgCRND8*, *APP^NL-F/NL-F^*, *APP^NL-G-F^*, *APP/PS1*, *3xTg-AD*, and *5xFAD* models. The results are summarised in Table 1.

***TgCRND8*** mice exhibit an overexpression of the human APP gene with two mutations associated with familial AD (FAD): the Swedish mutation (*KM670/671NL*) and the Indiana mutation (*V717F*) [33,34]. The retina of the *TgCRND8* mouse model has been examined using OCT in a single study. This utilised four male wild-type mice (*129/Sv*), aged 4–8 months, as a control group that was matched in terms of age and gender to the *TgCRND8* group [35].

The ***APP^NL-F/NL-F^*** model has been generated by knock-in (KI) insertion of humanised Aβ sequences containing FAD mutations, namely the Swedish (NL) and Beyreuther/Iberian (F) mutations [36]. There is only one study that investigates this model using OCT, in which male mice of 6, 9, 12, 15, 17, and 20 months of age were analysed and compared with age-matched WT (*C57BL/6J*) mice [37].

The ***APP^NL-G-F^*** KI mice were designed to increase Aβ_42_ levels without overexpressing the human APP gene. These mice carry Swedish, Arctic, and Iberian mutations in the APP gene [38]. The retina was analysed by OCT in only one study, which included female mice of 3, 6, 9, 12, and 18 months of age, with male mice included for the oldest age group in the WT group [39]

The ***APP/PS1 model (APPswe, PSEN1dE9)*** mice were generated by co-injecting two vectors controlled by the mouse prion promoter. In the APP gene, one vector carried the Swedish mutation (*KM670/671NL*), while the other vector carried the FAD-linked *PSEN1* gene lacking exon 9 (*dE9*) [33,40]. Regarding retinal analysis using OCT, only two studies have examined this model. Georgevsky et al. [41] conducted a longitudinal study from 3 to 12 months of age, comparing the retina with WT littermates (50 males and 50 females), while Harper et al. investigated the retina from 10 to 24 months of age, including transgenic mice and WT littermates (9 females and 6 males) [42].

The ***3xTg-AD*** model was generated by co-injecting two human genes carrying familial AD mutations [APP with the Swedish mutation (*KM670/671NL*) and MAPT with the P301L mutation] into homozygous *PSEN1* M146V KI mouse embryos. The expression of both transgenes is controlled by the mouse Thy1.2 promoter [33,40,43]. A total of six different studies have utilised OCT to analyse the retina in this model:

(i) Chiquita et al. [44] conducted a longitudinal analysis, examining male mice at 4, 8, 12, and 16 months of age and comparing them with age-matched WT mice (*C57BL/6J*);
(ii) Song et al. [45] employed female *3xTg-AD* mice and *B6129SF2/J* mice as WT group;
(iii) Gardner et al. [46] performed a cross-sectional study with 20 *3xTg-AD* mice (16 males and 4 females) at different ages (2, 4, 7 and 10 months), comparing them with 12 *C57BL/6J* mice (only males) as the control group;
(iv) Ferreira et al. [47] utilised the *3xTg-AD* model to compare the effects of disease and aging with a normative database of the *C57BL6/129S* model, using male mice at various ages (1, 2, 3, and 4 months);
(v) Guimarães et al. [48] analysed 60 male *3xTg-AD* mice and 57 male *C57BL6/129S* mice at different ages (1, 2, 3, 4, 8, 12, and 16 months) in a longitudinal study;
(vi) Batista et al. [49] examined both eyes of 144 male mice, including 57 *3xTg-AD* mice and 57 WT mice, at various ages (1, 2, 3, 4, 8, 12, and 16 months).

The ***5xFAD*** model was generated by co-expressing five mutations associated with AD in *APP/PS1* transgenic mice (Swedish (*KM670/671NL*), Florida (*I716V*), and London (*V717I*) variants in the *APP* gene, as well as *M146L* (A > C) and *L286V* mutations in the *PSEN1* gene) [33,50]. The *5xFAD* model has been utilised in three studies investigating retinal changes by OCT:

(i) Lim et al. [51] examined 32 *5xFAD* mice and 38 non-transgenic (WT) littermates with a *C57BL/6J* genetic background as controls at different ages (6, 12, and 17 months);
(ii) Kim et al. [22] used five transgenic mice and six *B6SJLF1/J WT* controls, all 6 months of age;
(iii) Matei et al. [52] employed 16 male transgenic mice and 16 male *C57BL/6J* WT controls, all of them 3 months old.

**Table 1:** Study design of the different OCT studies in AD murine models.

	AD Murine Model	WT	Laboratory	Age	Gender	N (Number)	Eye Selected
Buccarello et al., 2017 [35]	TgCRND8	129/Sv	Jackson Laboratories, USA	8 months	Male	4 AD/4 WT	Both
Salobrar-García et al., 2021 [37]	APP^NL-F/NL-F^	C57BL/6J	RIKEN Brain Science Institute, Saitama, Japan	6, 9, 12, 15, 17, and 20 months	Male	55 AD/41 WT	Left eye
Vandenabeele et al., 2021 [39]	APPN^L-G-F^	Not specify	Jackson Laboratories, USA	3, 6, 9, 12, and 18 months	Male (18 months)	Not specify	Not specify
					Female (all time points)		
Georgevsky et al., 2019 [41]	APP/PS1	Littermates	Australian phonemics facility (ANU Canberra)	3, 6, 9, and 12 months	Male and female (50:50)	70 (no more data)	Both
Harper et al., 2020 [42]	APP/PS1	Littermates	Jackson Laboratories, USA	10 to 24 months	AD (17F/7M)/WT (9F/6M)	24 AD/15 WT	Both
Chiquita et al., 2019 [44]	3xTg-AD	C57BL/6J	Not specify	4, 8, 12, and 16 months	Male	21 AD/22 WT	Both
Song et al., 2020 [45]	3xTg-AD	B6129SF2/J	Jackson Laboratories, USA	15–16 months	Female	Not specify	Ex vivo
Gardner et al., 2020 [46]	3xTg-AD	C57BL/6J	Not specify	2, 4, 7, and 10 months	AD (4F/16M)/WT (12M)	20 AD/12 WT	Left eye
Ferreira et al., 2021 [47]	3xTg-AD	C57BL6/129S	Not specify	1, 2, 3, 4 months	Male	57 AD/57 WT	Both
Guimarães et al., 2022 [48]	3xTg-AD	C57BL6/129S	Not specify	1, 2, 3, 4, 8, 12, and 16 months	Male	60 AD/57 WT	Both
Batista et al., 2023 [49]	3xTg-AD	C57BL6/129S	Not specify	1, 2, 3, 4, 8, 12, and 16 months	Male	57 AD/57 WT	Both
Lim et al., 2020 [51]	5xFAD	Littermates	Jackson Laboratories, USA	6, 12, and 17 months	Not specify	32 AD/38 WT	Not specify
Kim et al., 2021 [22]	5xFAD	B6SJLF1/J	Jackson Laboratories, USA	6 months	Female	5 AD/6 WT	One random
Matei et al., 2022 [52]	5xFAD	C57BL/6J	Jackson Laboratories, USA	3 months	Male	16 AD/16 WT	One random

WT: wild type, vs: versus, AD: Alzheimer’s disease.

### 3.2. Selection of the Analysed Eye

For a retinal analysis using OCT, the different studies have analysed either one eye or both in the animal models of AD (Table 1).

**Both eyes** were examined using OCT in: (i) the *APP/PS1* model in the studies conducted by Harper et al. (2020) [42] and Georgevsky et al. (2019) [41]; (ii) the *3xTg-AD* model in the works of Ferreira et al., Guimarães et al., Chiquita et al., and Batista et al. model [44,47,48,49]; and (iii) the *TgCRND8* model analysed by Buccarello et al. [35].

Only **one eye** has been used for OCT analysis in the following AD models: (i) the *3xTg-AD* model studied by Gardner and colleagues [46]; (ii) the *APP^NL−F/NL−F^* model utilised by Salobrar-Garcia et al. [37]; and (iii) the *5xFAD* model employed in the studies conducted by Kim et al. [22] and Matei et al. [52].

However, in three studies using OCT analysis, it is **not specified whether one eye or both** were examined. These studies include: (i) Vandenabeele’s work [39] using the *APP ^NL-G-F^* model; (ii) the study by Lim et al. [51] using the *5xFAD* model; and (iii) the study by Song et al. [45] in the *3xTg-AD* model. Notably, the latter study was conducted on enucleated eyes with the optic nerve, which were kept in a Ringer’s solution during the examination (non-in vivo study).

### 3.3. OCT Model

Several studies have utilised different retinal imaging microscopes and OCT models to analyse distinct mouse models with retinal degeneration. The results are summarised in Table 2.

The **Phoenix Micron IV retinal imaging microscope combined with the Phoenix OCT model** (Phoenix Research Laboratories, San Ramon, CA, USA) has been utilised in studies conducted by Buccarello et al. (2017), Chiquita et al. (2019), Ferreira et al. (2021), Guimarães et al. (2022), and Batista et al. (2023) [35,44,47,48,49]. This instrument features a superluminescent diode with a bandwidth of 160 nm and a central wavelength of 830 nm, providing an imaging depth of 1.4 mm and an axial resolution of 3 µm [47,48,49].

The **OCT SD-OCT Spectralis** from Heidelberg Engineering (Germany) was used in studies examining the *APP^NL−F/NL−F^* model [37] and the 5xFAD model [52]. Specifications include 820 nm wavelength of the laser light source, 870 nm wavelength of the SLD light source, 40,000 A-scans per second scanning speed, 3.9 μm digital axial resolution, 14 μm transverse resolution, 1.9 mm scanning depth, 30 × 30 degrees maximum field of view, 11 μm high speed mode isotropic resolution, and 5 μm isotropic high-resolution mode. The APP^NL−F/NL−F^ model utilised Heidelberg Eye Explorer v6.13 software, while the 5xFAD model employed version 1.9.10.0 of the same software.

The **Envisu R2200 OCT spectral domain** (Bioptigen Envisu R2200) and InVivoVue Diver 3.0.8 software (Bioptigen) were employed for the analysis of the *APP^NL-G-F^* model by Vandenabeele et al. [39]. In the analysis of 5xFAD mice, Lim et al. utilised the same OCT model along with the FIJI software (National Institutes of Health, Bethesda, MD, USA) (National Institutes of Health, Bethesda, MD, USA) [51].

The **spectral domain OCT from Wasatch Photonics** (USA) was utilised by Georgevski et al. (2019) in the *APP/PS1* animal model. This instrument features a field of view of >40°, a central wavelength of 800 nm, an axial resolution of 3.9 μm, a transverse resolution of 4 μm, and an image frequency of 20 Hz [41].

The **high-resolution polarisation-sensitive OCT (PS-OCT) system**, which is a modification of spectral-domain OCT, based on the system previously described by Fialová et al. (2016) [53] was used by Harper et al. (2020) [42] in the *APP/PS1* animal model. This system allows measurements of reflectivity, phase delay, fast axis orientation, and degree of polarisation uniformity. It features a multiplexed superluminescent diode (SLD, Superlum), with a central wavelength of 840 nm and a bandwidth of 100 nm, resulting in an axial resolution of 5.1 µm in the air [42,53].

An **angle-resolved low-coherence interferometry (a/LCI) system guided by OCT** (Wasatch Photonics, Spark, λ  =  830 nm, Δλ  =  155 nm, A-line rate = 40 kHz) was utilised by Song et al. (2020) to investigate the ex vivo retinas of the *3xTg-AD* model [45].

The **scatter angular resolution OCT (SAR-OCT)**, a specific subtype of angular resolution OCT was utilised by Gardner et al. (2020) in the study of the *3xTg-AD* model. This instrument uses a scanning source laser (1310 ± 70 nm; 100 kHz scanning speed) manufactured by Axsun, Inc. (Billerica, MA, USA) with an axial resolution of 11.7 µm in the air [46].

A **custom-built spectral-domain OCT system** was utilised by Kim et al. (2021) in the AD *5xFAD* model. This system incorporated a superluminescent diode (λ = 810 nm; Δλ = 100 nm; D-810-HP, Superlum, Carrigtwohill, County Cork, Ireland) as the light source. The axial and lateral resolutions were theoretically estimated to be 2.9 μm and 11 μm, respectively [22].

### 3.4. Image Acquisition Protocol and Retinal Sector Analysed

Using the **Phoenix Micron IV**, the following scanning protocols were employed:

(i) In the study by Buccarello et al., a two-dimensional scanning protocol (B-scan) with a circular diameter of 550 μm around the optic nerve head (ONH) was utilised [35].
(ii) Chiquita and colleagues employed two scanning protocols. One was a circular scanning protocol around the ONH, and the other was a linear scanning protocol consisting of one scan centred in the middle of the optic nerve head, along with six superior and six inferior scans [44].
(iii) Ferreira and collaborators used a scanning protocol with 512 B-scans, each containing 512 A-scans of 1024 pixels in length. All scans were performed horizontally centred with the optical disc and vertically positioned above it [47].
(iv) Guimarães et al. performed their analysis in a region located above the optic nerve. Their protocol involved 512 B-scans, with regularly spaced B-scans selected at intervals of five B-scans. This resulted in a total of 14,730 B-scans (512 × 1024 pixels). Each selected B-scan was cut out to obtain a region of 512 × 512 pixels, centred on the retina, with its location determined automatically [48].
(v) In the work by Batista et al., each data volume consisted of 512 B-scans, with each B-scan composed of 512 discrete samples of 1024 A-scans. The optic disc served as the reference point to select the retinal region in the image, which was aligned horizontally just above it [49].

The imaging acquisition protocols for the **Spectralis OCT** in the AD models were different and consisted of the following:

(i) Salobrar-García et al. analysed the retina by centring the optic nerve head, placing it in the centre of the scans with 61 horizontal scans. The thickness of the nerve fibre layer was analysed using an axonal ring scan around the optic nerve head. Additionally, these authors placed a +25 dp lens in front of the OCT camera and a contact lens that covered the mouse cornea, creating a uniform refractive surface [37].
(ii) Matei et al. analysed the retina by focusing on two adjacent regions to the optic nerve head: the nasal and temporal regions. In each region, 13 scans were performed, including one in the middle of the optic nerve head, six superior scans, and six inferior scans [52].

The scanning protocol for the **Envisu-R2200 OCT** varied according to the analysed studies. Lim et al. analysed retinal volumes (1.4 × 1.4 × 1.57 mm) centred on the optic nerve head, acquiring them with 200 horizontal B-scans, each composed of 1000 A-scans. These images had a lateral resolution of 7 µm and a depth resolution of 2.8 µm [51]. However, Vandenabeele utilised a different acquisition protocol, capturing images with 1000 A-scans and 100 B-scans, resulting in 1.4 × 1.4 mm images. This author analysed the retina at 16 equidistant points from the optic nerve [39].

In the imaging acquisition protocol for the **spectral domain OCT from Wasatch Photonics** used by Georgevsky et al., a 3 × 3 mm square fundus image was analysed. The ONH was centred in the image. In total, five B-scans (spaced 125 μm apart), with one centred on the ONH and two superior and two inferior parallel B-scans, were performed [41].

In the **high-resolution polarisation-sensitive OCT (PS-OCT) system** for image capture, the ONH was aligned with respect to the measurement scan. The field of view was 1 × 1 mm, and the scanning frequency was 83 kHz for each A-scan. In total, five B-scans, each composed of 512 scans at 400 points, were performed. These features resulted in images with a higher signal-to-noise ratio in terms of reflectivity, PS-OCT, and OCTA image production. The authors analysed the retina by tracing a ring around the ONH with an inner diameter of 500 µm and an outer diameter of 900 µm [42].

In the **angle-resolved low-coherence interferometry (a/LCI) system guided by OCT** used by Song et al., as the procedure was ex vivo, the acquisition had to take place within 20 minutes after eyeball extraction and had to be aligned using the ONH as a reference. In total, eight retinal locations were analysed, spaced 500 µm apart along the vertical and horizontal axis. At each location, a single horizontal and vertical OCT B-scan was performed [45].

Using the **scatter angular resolution OCT (SAR-OCT)**, Gardner and colleagues scanned the retina of AD mice in four 1.3 × 1.3 mm^2^ (512 × 512 A-scans) square sections (nasal, superior, temporal, and inferior), with the ONH positioned at one corner of the frontal view [54].

For the **custom-built spectral-domain OCT system** utilised by Kim et al., image acquisition was performed in all four quadrants of the retina, starting from ONH to fixate the central point (first dorsal and ventral, followed by nasal and temporal). Each OCT volume consisted of 4 × 600 × 600 A-scans [22].

These results are summarised in Table 2.

**Table 2:** OCT used, scan type, and retinal findings found in each study.

Authors and Year	OCT Device	Retina Protocol Scans	Software Segmentation and Retinal Layer/Complex Analysed	Retinal Findings	
				Retinal Thinning	Retinal Thickening
Buccarello et al., 2017 [35]	Phoenix Micron IV Image-guided OCT	Circular scan (diameter of 550 μm) around the ONH.	Insight software (Phoenix Research Laboratories). NFL/GCL, IRL, ORL, total retinal thickness.	RNFL-GCC (4 months)	-
Salobrar-García et al., 2021 [37]	SD-OCT Spectralis	61 horizontal scans centred in the ONH. RNFL Circular scan around the ONH	Heidelberg Eye Explorer software v6.13 Total retinal thickness and RNFL	Total retinal thickness (6, 9, 12, 15, and 20 months), RNFL (6, 20 months)	Total retinal thickness and RNFL (17 months)
Vandenabeele et al., 2021 [39]	Envisu R2200 spectral domain OCT	16 equidistant points from the optic nerve.	InVivoVue Diver 3.0.8 Total retinal thickness	12 until 18 months ONL	-
Georgevsky et al., 2019 [41]	OCT from Wasatch Photonics	3 × 3 mm square centred in the ONH. Five scans (spaced 125 μm apart), centred in the ONH and two superior and two inferior parallel scans.	A modified segmentation algorithm based on graph theory Inner retina (GCL-INL) Outer retina (OPL-RPE)	Inner retinal thickness at 9 months and outer retinal thickness at 12 months	-
Harper et al., 2020 [42]	High-resolution polarisation-sensitive OCT (PS-OCT) system	Five B-scans. Ring scans centred in the ONH at 500 µm and 900 µm of diameter.	Algorithm by Augustin et al., 2018 [55]. Total retinal thickness, inner retinal thickness, and outer retinal thickness of the ring surrounding the ONH	No changes	-
Chiquita et al., 2019 [44]	Phoenix Micron IV Image-guided OCT	Circular scan (diameter of 550 μm) around the ONH. One scan centred in ONH, six superior and six inferior scans	Insight software (Phoenix Research Laboratories). GCL + IPL, INL + OPL ONL, IS + OS, total retinal thickness	Total retinal thickness 4–16 months GCL + IPL, INL + OPL, at 4, 8, 12, and 16 months	ONL
Song et al., 2020 [45]	Angle-resolved low-coherence interferometry (a/LCI)	Ex vivo acquisition. Aligned using the ONH. Eight scans, spaced 500 µm apart along the vertical and horizontal axis.	Definition described by Srinivasan et al., 2014 [56] RNFL, OPL, and RPE	RNFL (15–16 months)	-
Gardner et al., 2020 [46]	Specific subtype of angular resolution OCT, termed scatter angular resolution OCT (SAR-OCT)	Four 1.3 × 1.3 mm^2^ (512 × 512 A-scans) square sections (nasal, superior, temporal, and inferior), with the ONH at one corner of the frontal view	Previously established algorithm Two regions: the superficial layers consisting of NFL, GCL, and IPL, and the ONL	Total retinal thickness (3, 5, 8 and 11 months) Inner layers (NFL + GCL + IPL) at 2 months and 7 months and ONL at 2 months	
Ferreira et al., 2021 [47]	Phoenix Micron IV Image-guided OCT	512 B-scans, each containing 512 A-scans of 1024 pixels in length. Centred in the ONH	Convolutional neural network (FCNN) following a U-type architecture RNFL-GCL, IPL, INL, OPL, ONL, IS, OS, and RPE.	Total retinal thickness (1, 2, 3 and 4 months)	RNFL-GCC
Guimarães et al., 2022 [48]	Phoenix Micron IV Image-guided OCT	512 B-scans, each containing 512 A-scans of 1024 pixels in length. Centred in the ONH	Not segmentation software Total retinal thickness	Not specified	Not specified
Batista et al., 2023 [49]	Phoenix Micron IV Image-guided OCT	512 B-scans, each containing 512 A-scans of 1024 pixels in length. Centred in the ONH	Deep learning approach based in Convolutional neural network (FCNN) following a U-type architecture RNFL-GCL, IPL, INL, OPL, ONL, IS, OS, and RPE.	Total retinal thickness (1, 2, 3, 4, 12 and 16 months) RNFL-GCL, IPL, ONL OS	OPL, IS and RPE
Lim et al., 2020 [51]	Envisu R2200 spectral domain OCT	Retinal volumes (1.4 × 1.4 × 1.57 mm) centred ONH, acquiring them with 200 horizontal B-scans, each composed of 1000 A-scans.	FIJI analysis software GCC (from RNFL to the IPL), total retinal thickness.	RNFL-GCC (6, 12 and 17 months)	IPL (6 months)
Kim et al., 2021 [22]	Custom-built spectral-domain OCT	In four quadrants of the retina, starting from ONH to fixate the central point (first dorsal and ventral, followed by nasal and temporal). Each OCT volume 4 × 600 × 600 A-scans	Manual segmentation RNFL, inner retina, outer retina, and total retinal thickness	Total retinal thickness, NFL, Innes retina thickness and Outer Retina thickness (6 months)	-
Matei et al., 2022 [52]	OCT SD-OCT Spectralis	13 scans, one centred in ONH, six superior and six inferior scans.	Heidelberg Eye Explorer software (1.9.10.0) Total retinal thickness	No changes	No changes

### 3.5. Layer Segmentation and Software Employed

In the studies of AD models using OCT, different procedures for retinal segmentation and various software were also utilised (Table 2):

In the study by Buccarello et al., the authors used Insight software (Phoenix Research Laboratories) and conducted four measurements: the nerve fibre layer/ganglion cell layer (NFL/GCL) complex, inner retinal layers (IRL), outer retinal layers (ORL), and total retinal thickness. The NFL/GCL complex was delimited from the inner limiting membrane to the boundary between the GCL and the inner plexiform layer (IPL). The total retinal thickness was delimited from the NFL/GCL complex to the outer edge of the outer segment of the photoreceptors (OS). IRL were demarcated from the IPL to the boundary between the inner nuclear layer (INL) and the outer plexiform layer (OPL). Outer retinal layers (ORL) were demarcated from the INL/OPL boundary to the OS [35].

Chiquita and colleagues employed InSight software (v.1, Voxeleron LLC—Image Analysis Solutions, Chabot Drive, CA, USA) for segmentation. The total retinal thickness was delimited from the inner limiting membrane to the retinal pigment epithelium (RPE). The segmentation conducted by the authors included the following components: the complex formed by the GCL + IPL layers, the INL + OPL layers, the outer nuclear layer (ONL), the inner segments (IS) + the OS, and the total retinal thickness [44].

In the study conducted by Ferreira, segmentation of the entire analysed volume was achieved by separating the 512 B-scans of the OCT. This enabled the authors to analyse eight different layers: RNFL-GCL complex, IPL, INL, OPL, ONL, IS, OS, and RPE. The segmentation was made possible by utilizing a fully convolutional neural network (FCNN) following a U-type architecture [57]. The results represent the average retinal thickness of the entire analysed eye region. Additionally, thickness maps consisting of 3 × 3 values were calculated by averaging 170 × 170 thickness values from the original maps. These maps were then resized to 510 × 510 pixels to ensure uniform block size across all regions [47].

Guimarães and colleagues did not segment the retina into layers, they only analysed the total retinal thickness [48].

In the 3xTg-AD longitudinal study, a deep learning approach to determine retinal layer segmentations was employed. They utilised a neural network model based on a fully convolutional architecture, following a U-type design [49]. The authors examined a total of eight structures, as previously described by Ferreira et al. [47]. The results were represented through retinal thickness maps, which were derived from the volumetrically segmented OCT data. To analyse the thickness variations within the image area, the authors considered nine regions of interest (ROIs) for each volume. These ROIs consisted of non-overlapping 3 × 3 blocks of 170 × 170 points. The original data were centrally cut to a volume of 510 × 510 to ensure consistent ROI size. Mean thickness values were calculated by averaging the 510 × 510 and 170 × 170 thickness values for the entire image area and each ROI, respectively [49].

The animal experimental module of the Spectralis OCT allows for automatic segmentation of all retinal layers, which can be displayed using concentric rings. In the study conducted by Salobrar-García, both the total retinal thickness and the thickness of the RNFL were analysed. All segmentations were manually corrected by an experienced researcher. For the total thickness analysis, the results were presented following the Early Treatment of Diabetic Retinopathy Study (ETDRS) format. A total of three concentric rings with a total diameter of 3 mm, centred on the optic nerve, were used. The central 1 mm ring was excluded from the analysis, leaving two rings: an inner ring with a 2 mm diameter and an outer ring with a 3 mm diameter. Additionally, these rings were divided into four quadrants: superior, inferior, nasal, and temporal. Regarding the analysis of RNFL thickness, the results were presented in six sectors: inferior-nasal, nasal, superonasal, superotemporal, temporal, and inferotemporal [37]. Using the same tool, Matei and colleagues analysed the temporal and nasal retina, but did not present the results in sectors. Instead, they only displayed the total thickness of the retina [52].

For the segmentation and analysis of Envisu-R2200 OCT (Bioptigen, Research Triangle, NC, USA) images, Lim and colleagues utilised the FIJI analysis software (National Institutes of Health, Bethesda, MD, USA). The segmentation of central B-scans was performed manually, delineating the ganglion cell complex (GCC) from the RNFL to the IPL, as well as determining the total retinal thickness. To calculate the thickness of the IPL, the differences between the GCC and RNFL were taken into consideration. The thickness of the outer retinal layers was obtained by subtracting the GCC from the total retinal thickness [51].

Similarly, using the same OCT device (Envisu R2200, Bioptigen, Research Triangle, NC, USA), Vandenabeele and colleagues employed InVivoVue Diver 3.0.8 software to measure retinal thickness. They averaged the measurements taken from 16 points around the optic nerve [39].

In the study conducted using the Spectral Domain OCT from Wasatch Photonics, Georgevsky and colleagues employed a modified version of the segmentation algorithm developed by Chiu et al., which is based on graph theory [58], for retinal segmentation. This algorithm facilitated the division of the retina into two regions: the inner retina (from GCL to INL) and the outer retina (from the OPL to the RPE). In cases where segmentation errors were observed, manual corrections were applied [41].

With PS-OCT, Harper et al. employed an algorithm previously used in a study by Augustin et al. [55] to segment the retina. The authors defined the total retinal thickness as the distance between the inner limiting membrane and the posterior surface of the RPE, with the posterior surface of the OPL serving as the boundary between the inner and outer retina. The study focused on assessing the total retinal thickness, inner retinal thickness, and outer retinal thickness of the ring surrounding the ONH. Additionally, the authors divided the ring horizontally and calculated the mean superior- and inferior-retinal thicknesses [42].

In the study conducted by Song et al. [45] using the angle-resolved low-coherence interferometry (a/LCI) system guided by OCT, the NFL, OPL, and RPE layers were manually segmented on the ex vivo OCT images following the definition described by Srinivasan [56].

In the study made by Gardner et al. using SAR-OCT, the retinal thickness of *3xTg-AD* mice was segmented into sublayers using a previously established algorithm. The sublayers were divided into two regions: the superficial layers consisting of NFL, GCL, and IPL, and the ONL [54].

In Kim et al.’s study, utilising a custom-built spectral-domain OCT system, measured the thickness of the NFL, inner retina, outer retina, and total retinal thickness in the 5xFAD model at a distance of approximately 600 µm from the ONH in each of the four quadrants. The segmentation of the sublayers was performed manually [22].

### 3.6. Retinal Findings in the Retina of Different Murine Models of AD

In the *TgCRND8* mouse model, Buccarello et al. demonstrated thinning of the RNFL/GCL complex at 4 months of age. However, the thickness of the other analysed complexes (IRL and ORL) did not exhibit significant differences compared to the control group [35].

In the *APP^NL-F/NL-F^* model, a significant thinning was observed in the global analysis and in the nasal and inferior temporal sectors of the RNFL at 6 months of age. In the same layer, thinning was found in the superotemporal, nasal, and global value sectors at 20 months of age. Changes were also observed in the total retinal thickness, with significant thinning in the temporal sector of the outer ring at 6 months, in the temporal sectors of the inner ring, and in the inferior sectors of both the inner and outer rings at 15 months. At 17 months of age, the authors reported a significant thickening in the inferior and nasal sectors of the inner and outer rings. Finally, at 20 months of age, a significant thinning was observed in the nasal, temporal, and inferior sectors of the inner ring, as well as in the superior and temporal sectors of the outer ring [37].

In the *APP^NL-G-F^* mouse model, Vandenabeele et al. did not observe any differences in total retinal thickness or in the RNFL/GCL, inner plexiform, or inner nuclear complex at any studied time. However, they did find a slight decrease in the thickness of the outer nuclear layer starting from 12 months to 18 months [39].

In the *APP/PS1* model, Georgevsky et al. observed a statistically significant decrease between AD model and WT mice at 9 months in the inner retinal thickness and at 12 months in the outer retinal thickness. Similarly, a significant thinning in outer retinal thickness was found in *APP/PS1* mice, while this thinning did not reach statistical significance in WT mice [41].

In the same animal model (*APP/PS1*), Harper et al. noticed a general trend of decreasing retinal thickness with age in both transgenic mice and WT controls. However, they did not find any statistically significant changes in retinal thickness between the transgenic and control groups for any of the retinal region [42].

In the 3xTg-AD model, the following results were found in the different analysed studies:

(i) The work by Chiquita et al. reported a statistically significant reduction in retinal thickness with respect to the WT group in all layers analysed except for the ONL, in which the authors showed a statistically significant increase in thickness in the *3xTgAD* group, with respect to the WT group [44].
(ii) Song et al. also found a statistically significant thinning of RNFL in AD mice compared to WT group. However, no statistically significant differences were observed in either OPL or RPE [45].
(iii) Gardner and colleagues observed statistically significant differences in total retinal thickness, as well as in the thickness of the most inner layers (NFL + GCL + IPL) and the ONL. Furthermore, the authors noted that the most consistent changes in thickness between the AD and control groups were observed in the central regions of the retina [46].
(iv) Ferreira et al. conducted a study on the aging *3xTgAD*, comparing it with a normative database of the *C57BL6/129S* model aged 1 to 4 months. The authors concluded that there were no statistically significant differences between the right and left eyes. However, when comparing the groups, a significant decrease in retinal thickness was observed, except in the RNFL-GCL complex, where the *3xTg-AD* mice showed thickening compared to the WT group. Additionally, no statistically significant differences were observed in the ONL. The authors further described that the most significant differences in thickness were found in the IPL, ONL, OPL, IS, RPE, and the total retina [47].
(v) Although Guimarães et al. did not provide detailed information on the specific alterations in retinal thickness, their findings clearly indicated that retinal aging exhibits distinct patterns between the AD group and the WT group [48].
(vi) Batista et al. conducted a longitudinal study to analyse retinal thickness in the *3xTg-AD* transgenic model compared to the WT group. The mean values of total retinal thickness showed no statistically significant differences between the left and right eyes, except for *3xTg-AD* mice at 1 month of age. Additionally, they found an increase in the total retinal thickness near the optic disc (from superior to inferior) in both eyes [49].

The authors also noticed a temporal-nasal asymmetry in both groups, with the temporal total retinal thickness being the thickest. When comparing the mean retinal thicknesses, statistically significant differences were found between WT and *3xTg-AD* mice at all ages. Overall, the retina of *3xTg-AD* mice was thinner than that of WT mice in both eyes throughout the study. However, the RNFL-GCL complex of *3xTg-AD* mice was thicker than that of WT mice starting from 8 months of age. Additionally, the OPL in *3xTg-AD* mice was consistently thinner compared to WT mice at all ages, except at 3 months.

Although both groups showed a decrease in total retinal thickness with age, indicating age-dependent thickness changes in most retinal layers/layer complexes, the behaviour of the individual layers differed. In the longitudinal study, the observed variations were the result of a balance between thinning of the RNFL-GCL, IPL, INL, ONL, and OS layers and thickening of the OPL, inner layer segment (ILS), and RPE layers. The INL and IPL layers exhibited the greatest decrease in thickness, while the RPE and ILS layers showed the greatest increase. However, the RNFL-GCL, OPL, ONL, and OS layers exhibited small to moderate thickness variations [49].

In the *5xFAD* model, Lim et al. demonstrated selective thinning of the inner retina. This model exhibited significant thinning of the RNFL at all tested ages compared to the WT group. The GCC also showed a significant reduction compared to the control group. However, when analysed separately, the IPL of *5xFAD* mice exhibited greater thickness than WT mice, particularly at 6 months of age [51].

In the same animal model (*5xFAD*), the study conducted by Kim et al. also found that total retinal thickness was significantly lower in *5xFAD* mice compared to WT mice. The analysis of retinal quadrants revealed a pattern of thinning, with the dorsal area of the inner retina exhibiting more pronounced retinal thinning compared to the ventral quadrant. Specifically, the NFL in the dorsal sector was notably thinner in *5xFAD* mice compared to the WT group. Additionally, the outer retina also exhibited thinning, which reached statistical significance in the temporal quadrant [22].

Finally, Matei and colleagues observed no differences in total retinal thickness in this animal model of AD (*5xFAD*) [52].

**Figure 3:**
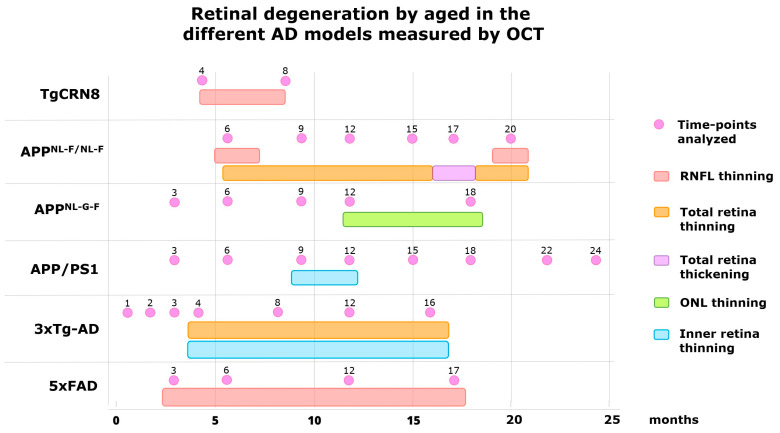
Retinal degeneration by age in the different AD models measured by OCT. These models have been analysed by: *TgCRND8* [35], *APP^NL-F/NL-F^* [37], *APP^NL-G-F^* [39], *APP/PS1* [41,42], *3xTg-AD* [46,47,48,49], and *5xFAD* [22,51,52].

These results are summarised in Table 2 and Figure 3.

## 4. Discussion

### 4.1. Differences among Animal Models of AD Used in OCT Studies

Although they are not the perfect experimental model for neurodegenerative diseases, mice are the most suitable animal for studying these conditions. Despite the aforementioned differences between human and rodent biology, mice exhibit more similarities than differences, and their retinas are organised into the same layers as human retinas. Additionally, due to their relatively short lifespan, mice are ideal for aging research [59,60] and are considered the preferred animal model for studying human aging [59].

In the reviewed literature, two studies utilised a first-generation murine model of AD (*APP/PS1*). This model is known for replicating key characteristics of the disease, such as Aβ deposition and neuroinflammation. However, it should be noted that this model, which involves the overexpression of *APP or APP/presenilin* 1 cDNA, can also exhibit artificial phenotypes due to mislocalisation or excessive production of *APP/PS1* [61]. Consequently, this model is associated with a high rate of premature mortality and neuronal cell death, without the accumulation of Tau or involvement of the inflammasome [62]. These limitations raise concerns regarding its suitability as an adequate model for studying AD and call into question the reliability of the obtained results.

A single OCT study examined the retinal changes in the *TgCRND8* model, which expresses a mutant form of *APP 695* with the Swedish (*KM670/671NL*) and Indiana (*V717F*) mutations in a hybrid *C3H/B6* genetic background. This model exhibits a substantial amount of Aβ accumulation at 3 months of age [35], followed by the presence of dense nuclear plaques and neuronal involvement at 5 months of age [34], thereby replicating features of early-onset or familial AD. Additionally, the model is characterised by early cognitive impairment, attributed to the overproduction of Aβ_42_, accumulation of pTau, and inflammation.

One of the most extensively studied animal models using OCT is the *3xTg-AD* model, characterised by the presence of three mutant human genes: presenilin 1 (*PS1 M146V*), amyloid precursor protein (*APP SWE*), and tau (*Tau P301L*). The model exhibits the appearance of Aβ oligomers between 2 and 6 months of age, with continuous accumulation observed between 12 and 20 months of age. Deposits become visible in the cerebral cortex at 6 months of age and progress to the hippocampus [43]. Additionally, pTau accumulation starts in the hippocampus at 12 months of age and extends to the cortex [63]. Synaptic dysfunction has also been observed in this model, occurring earlier than protein accumulation [43]. Importantly, this model mirrors the clinical features of advanced stages of AD.

Finally, the *5xFAD* model, which has been employed in three OCT studies, is characterised by the presence of *5xFAD* mutations (*APPSWE/FL/LON/PS1 M146L/L286V*). This model exhibits significant accumulations of Aβ_42_ in the cortex, leading to the formation of Aβ plaques and subsequent cognitive impairment [50]. It has been reported that at 6 months of age, this model also displays behavioural deficits [64], neuronal death [65], reduced capillary blood flow [66], and abnormal viscoelasticity in brain tissue [67].

All of the aforementioned models possess mutations in the presenilin gene (presenilin-1, presenilin-2, and amyloid protein precursor), resulting in early-onset AD. These autosomal dominant forms of AD, characterised by their early onset, are the least common in clinical settings and are less suitable for studying age-related forms of AD [68].

To avoid these problems, second-generation animal models were developed, including those with the Swedish (*KM670/671NL*) mutation [69], Beyreuther/Iberian (*I716F*) mutation [70], and with or without the Arctic mutation [71]. In OCT studies, the *APP^NL-G-F^* model was utilised in one study [39], and the *APP^NL-F/NL-F^* model was used in another [37]. The *APP^NL-G-F^* model is the most commonly employed in research due to its accelerated pathology development, being three times faster than the *APP^NL-F/NL-F^* model, which manifests pathology after 18 months, considered a lengthy duration for research purposes [38].

However, considering that the aim is to recapitulate certain aspects of neurodegenerative diseases such as AD as faithfully as possible, where age is the primary risk factor, it is somewhat incongruous to accelerate symptoms in young animals. This approach deviates from the natural characteristics of tissue when the disease develops. Moreover, given that mice aged 24 months correspond to approximately 80 years in humans [60], it would be more reasonable to analyse sporadic AD models at later stages rather than earlier ages with accelerate damage. Examining a model expressing the disease at 6 months would be analogous to studying a human with AD at 20 years of age.

### 4.2. Differences among the Variety of OCT Devices Used in the Studies of AD Animal Models

In the scientific literature, various OCT systems are employed. Firstly, there are commercially manufactured OCTs, such as Spectralis and Micron, which are produced by established companies. These OCT systems have undergone extensive development and refinement within the commercial sector, demonstrating that the measurements are reliable and repeatable. Secondly, there are self-built OCTs, which are developed by the research laboratories themselves. These custom-built OCT systems are typically tailored to specific research requirements and are often designed to explore novel imaging techniques or address unique scientific challenges.

OCT systems designed for human use have undergone extensive research to assess the accuracy of the segmentation algorithms employed. These algorithms are implemented within the OCT tool itself or developed by research laboratories [72,73]. The segmentation algorithms can measure various parameters, including total retinal thickness, as well as individual measurements of RNFL and GCL. Numerous studies have demonstrated the high clinical reliability of these OCT systems [74,75,76,77].

In mouse studies, distinguishing between the RNFL and the GCL using OCT is a challenge, leading to these layers being considered as a complex entity [78]. Furthermore, in many cases, OCT systems lack built-in segmentation software, need manual segmentation, or require the use of algorithms developed by the own researchers.

In the study of the TgCRND8 model, a Micron IV OCT combined with image-guided OCT was used [35]. This specific OCT model has also been employed in other studies analysing the *3xTg-AD* model [44,49], and it offers an axial resolution of 3 µm. However, in the investigation of the latter murine AD model *(3xTg-AD*) [79], the Micron IV OCT was replaced by the Phoenix Micron OCT2, which provides a higher resolution of 2 µm. Among the various studies examined, the Phoenix Micron OCT2 demonstrates the highest resolution capability.

In the study analysing the *5xFAD* murine model using the Heidelberg Spectralis OCT [52], the methodology description refers to two previous works, but neither of them provides detailed information about the procedure used for performing OCT. Additionally, it is not mentioned whether the imaging, obtained with the Spectralis OCT, was performed with a contact lens placed on the animals’ eyes. This aspect is important, as the contact lens is used to create a uniform refractive surface. Furthermore, there is no specification regarding the use of the animal module, as reported in the study of *APP^NL-F/NL-F^* AD murine model, which was conducted using the same instrument [37].

Spectral domain technology has been employed for the study of the *APP/PS1* murine model over time. A detailed description of the technical characteristics of its OCT, including field of analysis, wavelength, axial and transverse resolution, are provided in this study [41]. Spectral domain technology has also been used by other authors to study murine models of AD, such as [39,51] who have also utilised spectral domain technology. However, the Envisu-R2200 OCT used by these authors exhibits lower resolution values compared to the OCT utilised by Georgevsky. Additionally, Kim and colleagues [22] developed their own spectral domain OCT system, which has been employed in previous studies [80,81,82]. This system boasts a lateral resolution of 11 µm and an axial resolution of 2.9 µm.

On the other hand, among the reviewed papers, several research groups have employed modifications of OCT systems to assess additional tissue characteristics such as tissue reflectivity or polarisation state. In these instances, OCT serves as an imaging guide. For instance, the PS-OCT system used in the *APP/PS1* model study has an axial resolution of 3.8 µm [42], while for the *3xTg-AD* model study, SAR-OCT was used to quantify tissue scatter [46]. Similarly, for the study of the *3xTg-AD* model, OCT together with co-registered angle-resolved low-coherence interferometry (a/LCI) was also used to measure angle-resolved light scattering in the retina [45]. It is worth noting that these measurements were conducted on ex vivo transgenic mouse retinas, which may explain why the obtained results are less reliable compared to studies using live mice.

Only in the study of the *APP^NL-F/NL-F^* model, is it specified that the image capture was performed by means of a tracking system, which enables an analysis of the same area and helps prevent measurement errors or unintentional movements and allows a perfect follow up in the same animal [37].

### 4.3. Layer Segmentation and Software Employed in OCT Studies

Most authors have analysed the total retinal thickness and have also segmented it into inner and outer layers. However, one of the problems resides in the fact that each author delimits the internal complex and external complex in distinct manners (Table 2), which do not encompass the same layers within each of these complexes. Consequently, despite sharing the same denomination, they are not analysing the same entity. Thus, it is not possible to make direct comparisons between them.

Regarding the software used for segmentation, authors utilise the automatic software implemented in OCT due to the high complexity involved in distinguishing retinal layers. Additionally, some authors develop their own algorithms or convolutional networks for segmentation purposes. This approach eliminates the need for manual segmentation, which was only utilised in one study within this review. It is important to note that variations in segmentation methods can introduce minor errors in layer and complex demarcation, and in many instances, the accuracy of these methods remains unknown [83].

A previous study has compared the performances of two retinal layer segmentation algorithms with data obtained from manual segmentation and those obtained using the Heidelberg OCT Spectralis segmentation algorithm. In this previous study, the automated algorithms showed a good performance in segmenting the inner retinal layers, while the RPE delineation was less accurate [83].

Deep learning models and more specifically, Convolutional Neural Networks (CNNs) are recently being integrated. These new forms of data analysis are especially useful in retinal layer segmentation, as they can handle a large amount of information automatically [84].

### 4.4. Retinal Findings in OCT Studies

Taking into account the findings and comparing them across different studies, it is important to acknowledge the complexity involved due to variations in study durations and the use of different AD models. However, most of the studies consistently report a statistically significant thinning of the total retinal thickness. Additionally, four studies specifically indicate a thinning of the RNFL or the RNFL-GCL complex [35,45,49,51]. Conversely, thickening (which was only analysed in five studies) has been observed in the total thickness and certain inner and outer layers, but without uniformity or consistency [37,44,47,49,51].

When analysing the *3xTg-AD* and *5xFAD* models, it is interesting to observe that there are more differences within the results of the same model than when comparing the other models to each other. Therefore, in most of the models, a thinning of the inner retinal layers is observed, while only two of them show an involvement of the outer retinal layers. In the *3xTg-AD* and *5xFAD* models, both thinning and thickening of the inner and outer layers are observed. This could be attributed to the different analysis protocols used and the varying study durations. In a recent study analysing the *3xTg-AD* model, they describe the existence of a balance between thinning and thickening of the retinal layers [49].

The presence of significant variability among the different studies could be interpreted as varying degrees of neurodegeneration in each AD model.

Some of these studies have provided histological evidence that the observed decrease in peripapillary RNFL measured by OCT is attributed to the loss of retinal ganglion cells [35]. In addition, other authors have augmented their OCT-based structural investigations by incorporating immunohistochemical preparations to validate their findings. They demonstrated that the observed thickening in their model at 17 months of age was associated with astrocytic and microglial gliosis in the retinal [37].

In contrast to the homogeneity observed in human studies, where there is a consistent selection of study areas in the retina (macular area and peripapillary area) and a standardised representation of results using a protocolised pattern (macular ETDRS and sectors in the peripapillary RNFL), such uniformity is lacking in the analysis of OCT in AD murine models.

One limitation of the current study is the limited number of papers analysing the mouse retina using OCT. Up to the date of this review, only fourteen manuscripts were available, with six of them focusing on the same murine model of AD (*3xTg-AD*). Therefore, further research is needed to replicate these investigations and derive conclusive results regarding the retinal involvement in each animal model.

### 4.5. Future Perspectives Needed for the Use of OCT Retinal Analysis in Murine Models of AD

Because of the lack of standardised protocols for in vivo retinal OCT analysis in murine models of AD, analysis has recently been attempted in humans [85]; it would be necessary to determine certain recommendations for the use of retinal imaging as a potential biomarker of the disease.

Although it may seem utopian, it would be desirable for the OCT industry to manufacture devices with compatible technical specifications, having the same resolution, specificity, reproducibility, and standardised segmentation algorithms, so that measurements can be comparable. This will require collaboration between research groups and the industry.

In the case of devices manufactured internally by each working group, researchers should develop image-processing algorithms that convert their data into formats comparable to those of industry-created equipment, and to those of other research teams, allowing the data to be standardised and easily comparable.

Cooperation between researchers would also be useful for the development of a standardised database for different murine models and for different times of analysis. Currently only a few papers analyse the normal retinal thickness values of WT mice [86,87] and only the values in the AD *3xTg-AD* model have been standardised [47,49]. This would allow the exchange of data between research groups and create a comparative and accessible database similar to the AD Neuroimaging Initiative (ADNI) database [88].

It would also be necessary to develop a protocol for capturing OCT images. The first thing that should be unified is which area of the retina should be analysed. It should be considered that the anatomy of the murine retina is very different from the human retinal anatomy and therefore the anatomical areas to be analysed should be fixed.

Image quality control mechanisms are essential for the use of OCT as AD biomarkers and multicentre assays. Minimum standards should therefore be set to consider the images to be of sufficient quality for further processing.

Regarding segmentation, it would be essential to perform a uniform segmentation of the retinal layers and to establish the limits of the sub-layers or complexes. For this, it is essential that the measuring OCT devices have the same spatial resolution. Standard guidelines for interpretation, classification, and the reporting of results are needed. Patterns of retinal involvement should be developed for each of the murine models of AD. In addition, these patterns could be useful for classification between pathological and healthy retinas, as well as different neurodegenerative pathologies.

## 5. Conclusions

The utilisation of OCT for retinal analysis in murine models of AD represents significant progress in the research of retinal involvement in the pathophysiology of the disease. However, the number of studies conducted thus far is limited, and there is a lack of consensus regarding examination criteria and the specific areas analysed. Therefore, it is crucial to establish a standardised protocol for the analysis and representation of results in order to facilitate comparability and enhance the validity of findings.

## Data Availability

Data are available on request from the corresponding author.
